# Gender Differences in Emotional Response: Inconsistency between Experience and Expressivity

**DOI:** 10.1371/journal.pone.0158666

**Published:** 2016-06-30

**Authors:** Yaling Deng, Lei Chang, Meng Yang, Meng Huo, Renlai Zhou

**Affiliations:** 1 State Key Laboratory of Cognitive Neuroscience and Learning, Beijing Normal University, Beijing, China; 2 Department of Psychology, School of Social and Behavior Sciences, Nanjing University, Nanjing, China; 3 Research Center of Emotion Regulation, Beijing Normal University, Beijing, China; 4 Department of Psychology, University of Macau, Taipa, Macau S.A.R., China; 5 Department of Human Development and Family Sciences, University of Texas at Austin, Austin, Texas, United States of America; University of Würzburg, GERMANY

## Abstract

The present study investigated gender differences in both emotional experience and expressivity. Heart rate (HR) was recorded as an indicator of emotional experience while the participants watched 16 video clips that induced eight types of emotion (sadness, anger, horror, disgust, neutrality, amusement, surprise, and pleasure). We also asked the participants to report valence, arousal, and motivation as indicators of emotional expressivity. Overall, the results revealed gender differences in emotional experience and emotional expressivity. When watching videos that induced anger, amusement, and pleasure, men showed larger decreases in HR, whereas women reported higher levels of arousal. There was no gender difference in HR when the participants watched videos that induced horror and disgust, but women reported lower valence, higher arousal, and stronger avoidance motivation than did men. Finally, no gender difference was observed in sadness or surprise, although there was one exception—women reported higher arousal when watching videos that induced sadness. The findings suggest that, when watching videos that induce an emotional response, men often have more intense emotional experiences, whereas women have higher emotional expressivity, particularly for negative emotions. In addition, gender differences depend on the specific emotion type but not the valence.

## Introduction

A common stereotype in both Western and Eastern cultures suggests that women are more emotional than men, particularly when responding to negative emotions [1]. Despite substantial efforts in gender differences in emotional responses over the past several decades, no consensus as to whether women are more emotional than men has been reached. Gard et al. [1] stated that researchers should consider both emotional experience and emotional expressivity when investigating gender differences in emotional responses. Emotional experience refers primarily to an individual's physiological arousal evoked by external stimuli, and emotional expressivity is the external expression of subjective experience. Kret et al. [2] agreed with this notion and further noted the importance of addressing specific types of emotion when investigating gender differences in emotional responses. Therefore, the present study investigated gender differences in both emotional experience and emotional expressivity and considered eight emotion types.

It remains unclear whether gender differences exist in emotional experience. Numerous studies have shown that, compared with men, women usually experience more frequent and stronger negative emotions [3,4]. This may explain why more women are more prone to mood disorders [4–7]. Gohier et al. [8] adopted a priming paradigm and found that negative stimuli reduce the priming effects on women. They explained that women are more sensitive to negative stimuli, and this heightened sensitivity interferes with their processing of negative stimuli. Electrophysiological studies have shown that women exhibit greater galvanic skin response and an elevated heart rate (HR) when watching movies that induce feelings of sadness, and their HR is also elevated in response to movies that induce feelings of disgust [3]. Bradley et al. studied startle reflex reactions and revealed that women exhibit a stronger response to negative stimuli [4]. However, an increasing number of studies have shown that men exhibit more intense emotional reactions, particularly to stimuli that are perceived to be threatening [2] or erotic [9].

In addition, many studies have suggested there are no gender differences in emotional experience [6, 10, 11]. Electrophysiological studies have shown that HR is lowere when people view emotion-inducing pictures, but this variance in HR does not differ between genders [10]. The same finding has been reported by studies investigating emotional responses to movies [11]. Another two studies on startle reflex reactions have found that no gender differences exist when the participants watched negative stimuli [6, 12]. Similarly, Fischer and Manstead [13] stated that despite the large number of studies that have confirmed gender differences in emotional experience, these differences were smaller than expected, with almost no differences being reflected in the observed behaviors of participants.

Regarding gender differences in emotional expressivity, no consensus has been reached. Many studies have used subjective evaluations as indicators of emotional expressivity, fingding that women often report a more intense emotional response regardless of valence [7, 14–16]. For example, one study found that, compared with men, women rated negative stimuli with higher arousal and rated neutral stimuli more positively [17]. Other studies have also shown that women rated dynamic anger and pleasure emotions as more intense than static emotions, but men rated only anger as more intense [18]. Furthermore, a series of results indicated that compared to men, women had a greater degree of differentiation in emotional expressivity on both positive and negative emotions [1]. However, several studies have also shown that there were no gender differences existed in subjective evaluations when the participants viewed pictures [19], faces [20], or movies [11] that induced emotional responses.

In summary, gender differences in emotional responses remain unclear. We considered two primary reasons for this. First, studies have confused the two concepts of emotional experience and emotional expressivity when investigating emotional responses. Some researchers have considered emotional experience as an indicator of emotional response, whereas others have considered emotional expressivity to be the indicator. However, emotional responses are multichannel and multisystem phenomena including physiological responses, subjective feelings, and behavior. The study of emotional responses should be based on the same reaction system (automatic versus reflective) to make a direct comparison [21]. Physiological responses and subjective evaluations belong to different reaction systems, namely the automatic and reflective systems, respectively [21]. The present study clearly distinguished the two aspects of emotional responses. The results of physiological reactions were considered indicators of emotional experience, whereas the results of subjective evaluations were considered indicators of emotional expressivity. We examined the gender differences in emotional responses, including both emotional experience and emotional expressivity. Second, some previous studies have considered the valence (positive, negative, neutral) of emotions, whereas others have specified several types of emotion, rending it difficult to directly compare the findings of such studies. Emotional content can provide more crucial information than valence can [11]. An increasing number of researchers believe that gender differences should depend on the specific type of emotion [2]. Thus, analyzing each specific type of emotion separately is imperative.

The present study investigated gender differences in emotional responses in different types of emotion including both emotional experience (by using objective physiological indicators) and emotional expressivity (by using a subjective report). We hypothesized that gender differences exist in emotional experience and emotional expressivity. We also hypothesized that gender differences in emotional experience and emotional expressivity may depend on specific emotions but not valence.

## Methods

### Ethics

The experimental procedures were approved by the Institutional Review Board of the State Key Laboratory of Cognitive Neurosciences and Learning of Beijing Normal University. All the participants signed an informed consent before participating.

### Participants

We recruited volunteers at Beijing Normal University through advertisements. Given that people with depression and alexithymia cannot accurately express their emotions or complete the emotion-elicited experiment [22], we used the Beck Depression Inventory (BDI) [23] and Toronto Alexithymia Scale (TAS-20) [24] to screen only those who did not show depressive tendencies (BDI≤4 points) and were able to express their emotional feelings (TAS-20≤66 points). A total of 110 volunteers agreed to participate in this study, but only 83 were screened in based on the inclusion criteria. We further excluded four participants with incomplete data because of mechanical failure. The final sample comprised 79 (31 men, 48 women; *M*_age_ = 20.89 years) participants. They were all healthy and right-handed, and their vision was normal or corrected normal.

### Material

For effective emotion induction, the current study used video clips that induced emotional responses [3]. The selected types of emotion were categorized according to Gross [25]: sadness, anger, horror, disgust, neutrality, surprise, amusement, and pleasure. For each emotion type, two video clips that were between 60 and 245 seconds in length were selected. A previous study has found that these lengths provided sufficient time for recording physiological responses [26].

### Recording

In the present study, we measured emotional responses with both subjective and objective measures. We used the paper version of the Self-Assessment Manikin [27] for the participants to rate the valence, arousal and motivation, including their ratings of (1) how happy or unhappy they were, (2) how calm or aroused they were, and (3) their desire to approach or avoid the scenes in the video clips. A 9-point Likert scale ranging from 1 (*not at all*) to 9 (*very much*) was used. We selected Heart Rate (HR) as the physiological response because it is currently the most common autonomic nervous system marker of emotional processing [28]. The HR was collected on a BIOPAC MP150 system with the AcqKnowledge 4.0 (BIOPAC Systems Inc). HR was assessed using a three-lead ECG, with a lead II configuration and analyzed offline using AcqKnowledge 4.0 software (BIOPAC Systems Inc).

### Procedure

The participants watched the video clips in a room under appropriate lighting condition. They were seated individually and directly in front of the screen. The video clips were displayed on a 14-inch computer screen. Four experimenters, who were trained psychology graduate students at Beijing Normal University, described to the participants that the study purpose was to learn more about emotions. The participants were informed that they should watch the videos carefully, but could look away or shut their eyes if they found the videos too distressing. They were also told that they could stop the experiment at any time if they felt uncomfortable. After the experiment was completed, the experimenters explained that this study was aimed at examining gender differences in emotional responses. Each participant received 90 RMB as compensation.

Prior to viewing each video, the participants were shown a blank screen for 30 seconds to allow time for them to clear their minds of all thoughts, feelings, and memories. Subsequently, they were asked to remain still or not make any strong movements while watching the video to ensure the quality of HR data. After each video, they were asked to complete the self-report inventory and clear their mind for 30 seconds before watching the next video. Each participant viewed 16 video clips (two video clips per emotion type). The order of the videos was organized so that: (1) no two videos targeting the same emotion were shown consecutively; (2) no more than three videos of a particular valence (negative or positive) were shown consecutively. All the videos were counterbalanced for different participants.

### Data Analysis

To eliminate gender differences in the response patterns, we used the results of neutral videos as a control standard. The dependent variable was the difference-value (D-value), which represents the score for other types of emotion minus that for neutrality. The scores were calculated before performing an analysis of variance (ANOVA). This method was adopted from a previous study [29]. Four separate mixed ANOVA tests of gender (men and women) and emotion type (sadness, anger, amusement, surprise, horror, disgust, and pleasure) were performed for the valence, arousal, motivation, and HR. Emotion type was within-subjects factor. We also calculated the correlation between the subjective scale scores and physiological responses. All the multiple pairwise comparisons were performed using Bonferroni’s correction. The uncorrected level of statistical significance was set at *p* < .05. We performed 28 (4 dependent variables × 7 emotion types) multiple pairwise comparisons at most, accordingly, the corrected alpha value was set at *p*<0.002 (0.05/28 = 0.002). Data analyses were performed using IBM SPSS Statistics 20.0 (IBM).

## Results

### Correlation between the subjective assessment scores and physiological responses

We calculated the correlation between the subjective assessment scores (emotional expressivity) and physiological responses (emotional experience). The results revealed a nonsignificant correlation between the subjective assessment scores and physiological responses in all types of emotion in men or women. We considered the subjective assessment scores and physiological responses to be inconsistent, meaning the emotional expressivity and emotional experience were inconsistent. Further analyses were performed to test whether the gender differences in emotional expressivity and emotional experience depend on the emotion types.

### Gender differences in emotional experience

**Fig 1:**
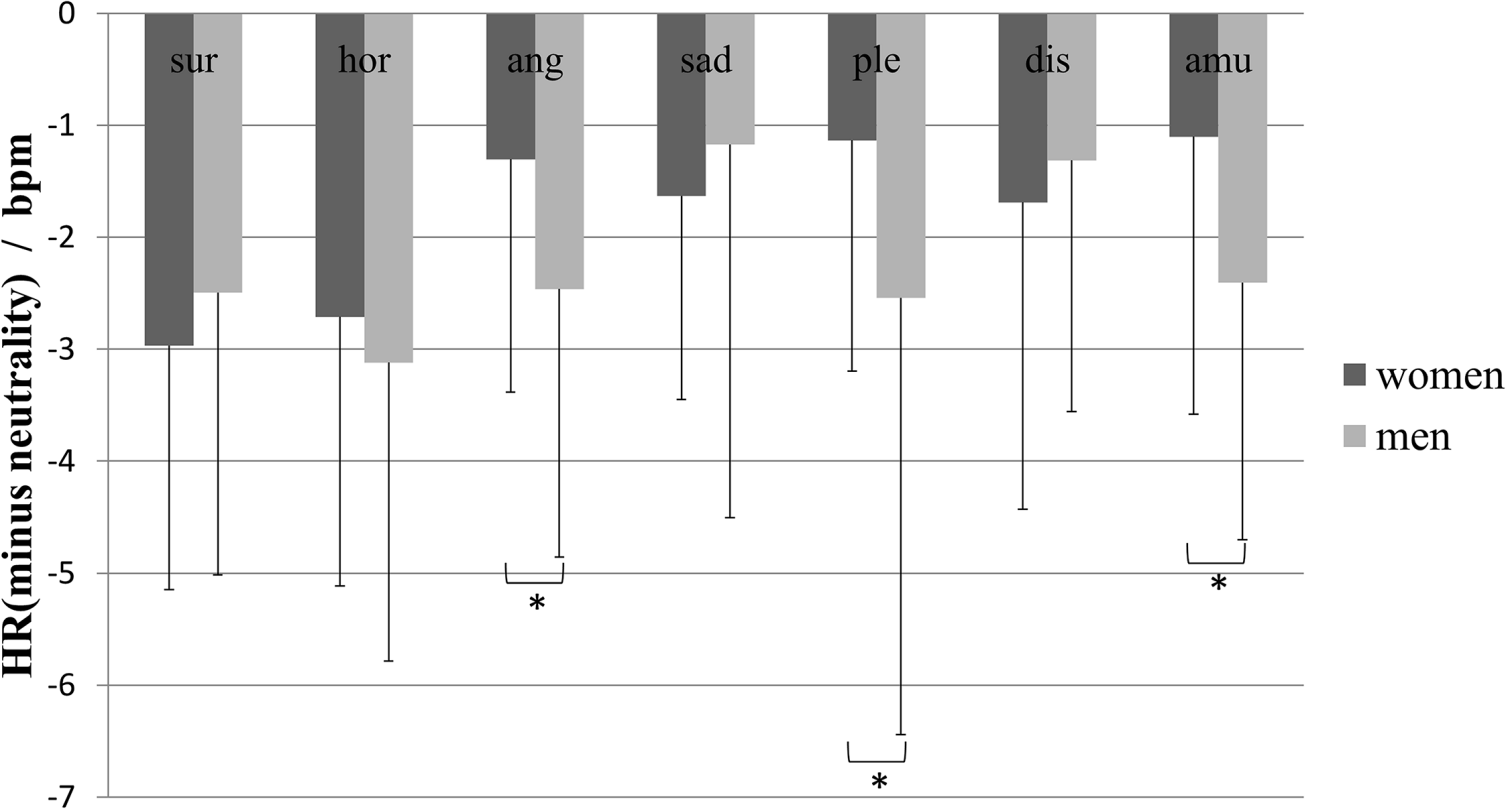
The D-value of HR between each type of emotion-inducing videos and those that induced neutrality of men and women. Statistical significance: *p<.002. Unless marked with an asterisk, no significant differences between these groups were found. Dis: disgust, hor: horror, ang: anger, sur: surprise, amu: amusement, ple: pleasure.

Mixed ANOVA of gender and emotion type showed a significant main effect of emotion type (*F*_*(6*,*456)*_ = 5.783, *p* < .001, *η*^*2*^ = .071), a nonsignificant main effect of gender (*F*_*(1*,*76)*_ = 1.360, *p* = .247, *η*^*2*^ = .018), and a significant interaction of gender and emotion type (*F*_*(6*,*456)*_ = 3.129, *p* = .005, *η*^*2*^ = .040). The results of simple effects analysis are shown in Fig 1. The dependent variable was the D-value of HR between each type of emotion-inducing videos and those that induced neutrality. The figure shows that the HR declined when the participants watched emotion-inducing videos compared with when they watched neutral videos for both men and women. Regarding the gender differences in HR, women exhibited a significantly smaller decline in HR while watching videos that induced anger (*M* = -1.305, *SD* = 2.080 versus *M* = -2.464, *SD* = 2.394; *p* < .002), pleasure (*M* = -1.134, *SD* = 2.062 versus *M* = -2.540, *SD* = 3.904; *p* < .002), and amusement (*M* = -1.103, *SD* = 2.477 versus *M* = -2.405, *SD* = 2.299; *p* < .002).

### Gender differences in emotional expressivity

**Fig 2:**
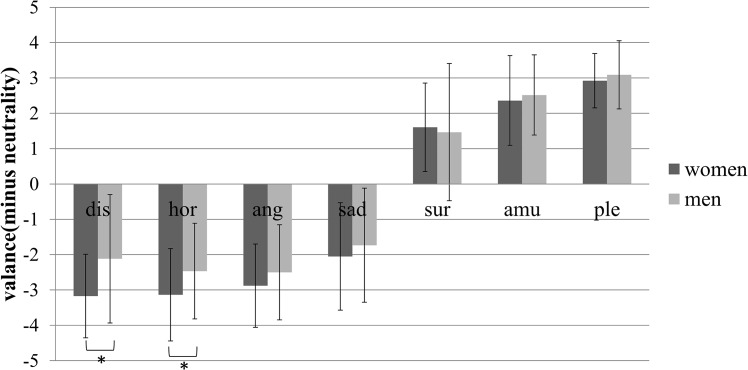
The D-value of the valence between each type of emotion-inducing videos and the videos inducing neutrality of men and women. Statistical significance: *p<.002. Unless marked with an asterisk, no significant differences between these groups were found. Dis: disgust, hor: horror, ang: anger, sur: surprise, amu: amusement, ple: pleasure.

Regarding the valence, mixed ANOVA of gender and emotion type showed a significant main effect of emotion type (*F*_*(6*,*462)*_ = 494.659, *p* < .001, *η*^*2*^ = .865), a nonsignificant main effect of gender (*F*_*(1*,*77)*_ = 2.962, *p* = .089, *η*^*2*^ = .037), and a significant interaction of gender and emotion type (*F*_*(6*,*462)*_ = 2.692, *p* < .05, *η*^*2*^ = .034). Further simple effects analysis is shown in Fig 2. The dependent variable was the D-value of the valence between each type of emotion-inducing videos and the videos inducing neutrality. The figure shows that the valence of negative emotions (disgust, horror, anger, and sadness) was significantly lower than that of neutrality, and the valence of positive emotions (surprise, amusement, and pleasure) was significantly higher than that of neutrality (all *p* < .002). Among the seven emotions, gender differences were evidenced by significantly lower valence ratings by women, but only for the disgust-inducing videos (*M* = -3.171, *SD* = 1.182 versus *M* = -2.117, *SD* = 1.815; *p* < .002) and the horror-inducing videos (*M* = -3.135, *SD* = 1.307 versus *M* = -2.466, *SD* = 1.354; *p* < .002).

**Fig 3:**
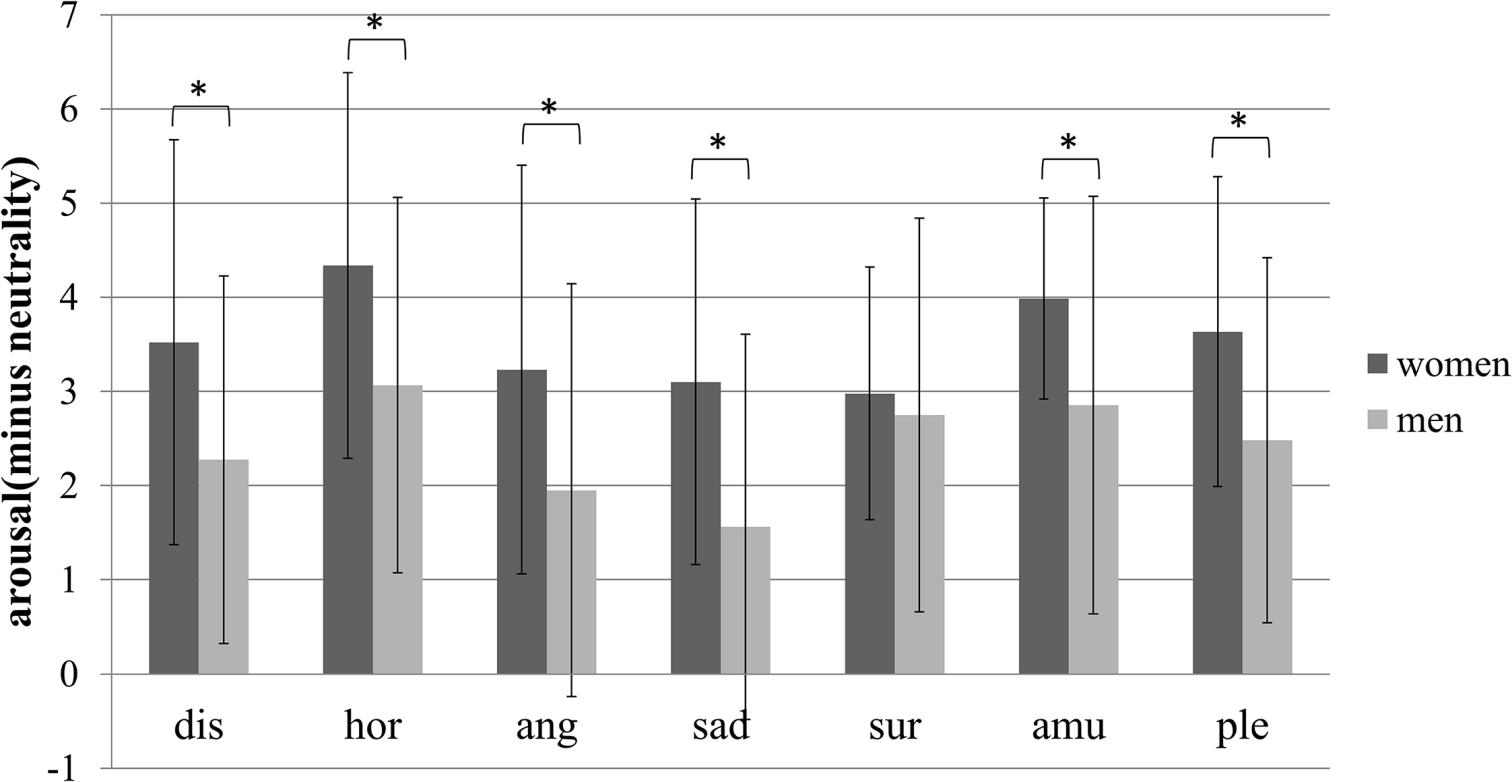
The D-value of arousal between each type of emotion-inducing videos and those that induced neutrality of men and women. Statistical significance: *p<.002. Unless marked with an asterisk, no significant differences between these groups were found. Dis: disgust, hor: horror, ang: anger, sur: surprise, amu: amusement, ple: pleasure.

As for arousal, mixed ANOVA of gender and emotion type showed a significant main effect of emotion type (*F*_*(6*,*462)*_ = 8.359, *p* < .001, *η*^*2*^ = .098), a significant main effect of gender (*F*_*(1*,*77)*_ = 12.010, *p* < .001, *η*^*2*^ = .135), and a nonsignificant interaction of gender and emotion type (*F*_*(6*,*462)*_ = 1.652, *p* = .131, *η*^*2*^ = .021). Despite the nonsignificant interaction effect, we also performed a simple effects analysis. Fig 3 shows the D-value of arousal between each type of emotion-inducing videos and those that induced neutrality. The figure shows that the arousal stimulated by the emotion-inducing videos was significantly higher than stimulated by those inducing neutrality (all *p* < .002). The results also showed that women reported a higher arousal value than did men on all emotion types (all *p* < .002) except the surprise emotion (*p*>.002).

**Fig 4:**
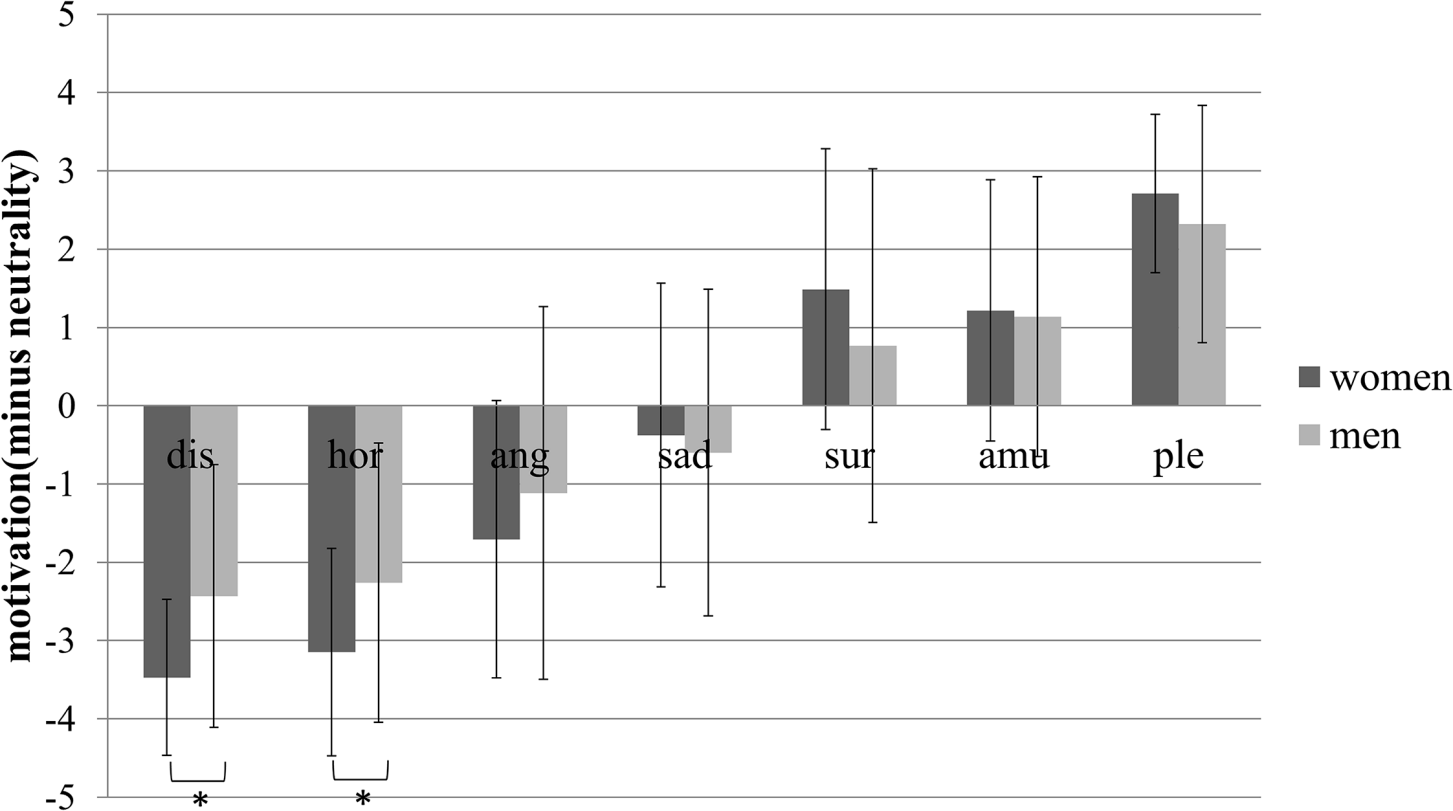
The D-value of motivation between each type of emotion-inducing videos and those inducing neutrality of men and women. Statistical significance: *p<.002. Unless marked with an asterisk, no significant differences between these groups were found. Dis: disgust, hor: horror, ang: anger, sur: surprise, amu: amusement, ple: pleasure.

Regarding motivation, mixed ANOVA of gender and emotion type revealed a significant main effect of emotion type (*F*_*(6*,*462)*_ = 157.721, *p* < .001, *η*^*2*^ = .672), a nonsignificant main effect of gender (*F*_*(1*,*77)*_ = .394, *p* = .532, *η*^*2*^ = .005), and a significant interaction of gender and emotion type (*F*_*(6*,*462)*_ = 4.174, *p* < .001, *η*^*2*^ = .051). Further simple effects analysis is shown in Fig 4. The dependent variable was the D-value of motivation between each type of emotion-inducing videos and those inducing neutrality. The figure shows that the emotions of sadness, disgust, horror, and anger induced avoidance motivation compared with neutrality, and the emotions of surprise, amusement, and pleasure induced approach motivation compared with neutrality. Gender differences were evidenced by women exhibiting higher avoidance motivation for the horror-inducing videos (*M* = -3.145, *SD* = 1.32 versus *M* = -2.259, *SD* = 1.782; *p* < .002) and disgust-inducing videos (*M* = -3.471, *SD* = .994 versus *M* = -2.431, *SD* = 1.677; *p* < .002).

Table 1 summarizes the gender differences for emotional expressivity and emotional experience for each type of emotion.

**Table 1:** Gender differences for emotional expressivity and emotional experience.

	emotional expressivity			emotional experience
	Valence	Arousal	Motivation	Heart rate
**anger**	-	women>men	-	decline: men>women
**amusement**	-	women>men	-	decline: men>women
**pleasure**	-	women>men	-	decline: men>women
**horror**	women<men	women>men	avoidance: women>men	-
**disgust**	women<men	women>men	avoidance: women>men	-
**sadness**	-	women>men	-	-
**surprise**	-	-	-	-

“-” means no gender difference.

## Discussion

This study extends previous studies on gender differences in emotional responses evaluated according to emotional experience and emotional expressivity. We observed gender differences in emotional responses and found that they depend on specific emotion types but not valence. Women show relatively stronger emotional expressivity, whereas men have stronger emotional experiences with angry and positive stimuli.

The self-report results are identical to those reported in several previous studies. Women often report more intense emotional responses [25], particularly for negative emotions [30]. Women in the present study reported higher arousal compared with men on most emotion types. Women also reported lower valence, higher arousal, and stronger avoidance motivation on disgust and horror emotions. The physiological results, such as the decline in HR while watching emotional stimulus, are also highly similar to those reported in previous studies [4,10,11]. This decline reflects the orientation, sustained attention, and action preparation of the viewers [10]. However, regardless of the valence, men exhibited a larger decline in HR than did women.

In contrast to our results, Fernández et al. [3] reported a positive correlation between HR and arousal. In the present study, we found no correlation between the subjective assessment scores and physiological responses, regardless of the type of emotion or the gender of the participant. According to Evers et al. [21], emotional experience and emotional expressivity belong to different reaction systems. The inconsistency between these two aspects is understandable. In support of the conclusion of Evers et al., we found similar inconsistencies in our results. Kret et al. [2] agreed with this notion and noted that, even in the presence of gender differences in emotion recognition, facial expressions, and subjective assessment, this does not imply that gender differences exist in emotional experience.

The present study also shows that gender differences depend on the emotion type but not the valence. First, for the negative emotions, gender differences were observed in horror and disgust. However, although men and women had the same emotional experience, women had stronger emotional expressivity, as evidenced by their lower valence scores, higher arousal, and stronger avoidance motivation. This finding is consistent with Codispoti et al. [10]. For the anger emotion, we found that men had stronger emotional experiences (e.g., a larger decline in HR), whereas women had stronger emotional expressivity (e.g., higher reported arousal). Previous studies have also found that men had a more intense physiological response to anger-inducing stimuli [2]. Regarding the sadness emotion, we observed no gender difference in emotional experience, although women reported a higher level of arousal.

The aforementioned emotions are all negative emotions, but their patterns in gender differences differ. Most previous studies have considered only the valence of emotions, and few have distinguished the content of the emotion. This finding might explain why no consensus has been reached [2,4–7,19]. Although many studies have argued that women are more sensitive to negative stimuli, many other studies have found that men are more sensitive to threat or sexual stimuli [2]. Our study also shows that men have stronger physiological responses on anger.

Second, for the positive emotions, the results show that men have a larger decline in HR while watching amusement- and pleasure-inducing videos, whereas women have higher levels of arousal. This is consistent with the findings of a previous study that showed that men had stronger physiological responses when watching positive videos [9]. However, this is inconsistent with Codispoti et al., who found no gender differences in participants while watching pleasant films [10]. This inconsistency may be due to the different stimuli used. Codispoti et al. [10] used a scene of sexual intercourse as a stimulus of pleasure, but we used scenes such as an enjoyable tour with family members.

According to these results, the present study does not support the widely accepted notion that women are more emotional than men [13] or that women were more easily affected by emotions [20], but our results support that women often report more intense feelings [31]. We suggest that gender differences in emotional responses should be considered according to different types of emotion, and there should be a distinction between the emotional experience and emotional expressivity.

There are numerous possible theoretical explanations of the reasons for gender differences, including differences in brain structures and sex hormones [2]. Here, our discussion is focused more on the reasons for inconsistencies in gender differences between emotional experience and expressivity, particularly regarding why women report more intense emotions.

First, it may be reasonable to speculate that the inconsistencies are attributable to human survival in terms of evolution and adaptation. Vigil [32] indicated that gender differences are the result of people adapting to social structures. Men usually lived in their own tribe, whereas women often marry to other tribes and assume the role of taking care of children. In competitive environments, women must rely on the support of the tribe to ensure that their children receive better care. In stressful situations, women require company and support more than men do [33]. Thus, for women, identifying the emotions and expressions of others and to expressing themselves quickly and effectively are critical. Women often persuade others to help them by expressing strong emotions. For men, their main roles are hunting and protecting family members. Therefore, they must be sensitive to the threat stimuli, including anger, fear, and similar emotions. Gender differences in emotions may have evolved from the need to adapt.

Second, although men experience strong emotions, gender stereotypes may have made them unwilling to express themselves honestly. Studies have found that gender stereotypes were likely to lead to the observed gender differences in emotional reactions untrue [34, 35]. Men are likely to assess emotions according to social expectations. Social stereotypes require men to be brave and calm, particularly in the face of anger and horror emotions. Thus, even when men experience very strong physiological arousal, they might not report experiencing strong emotions and their assessment might be relatively conservative to make others think they have not been influenced strongly [36]. In the present study, men had stronger emotional experience on the anger emotion, but they gave this emotion a lower rating. Regarding the horror emotion, men experienced the same extent of horror as women did, but men reported a lower rating. This may be another reason as to why gender differences were inconsistent between emotional experience and emotional expressivity.

Finally, the participants in this study may have regulated their emotions while watching the emotional videos. Although they were asked to feel their emotions, the possibility of emotion regulation cannot be excluded. Studies have found that men and women often use different strategies to regulate their emotions [37]. Emotional expressivity is reflected in the results of emotional experience after emotional regulation. The gender differences in emotional responses (particularly emotional expressivity) may be due to the gender differences in emotional regulation. Some studies have indicated that women have greater up-regulation of emotional responses to negative stimuli, which means that they often compound negative emotions [38]. The results of the present study might support this. Even when the women did not experience a particularly strong negative emotion, they might have regulated their emotions, interpreting them as more negative, which might explain why their expressivity was more intense for emotions such as anger, horror and disgust.

The present study explored gender differences in emotional experience and emotional expressivity for specific types of emotion in more detail than previous studies have. However, several limitations cannot be ignored. First, we discussed physical gender, not psychological or social gender. With the development of society, increasingly more women participate in social competition. Such social changes may affect the development of social gender roles, thereby affecting emotional responses. Second, the numbers of male and female participants differed considerably (women: men = 1.5: 1), resulting in two markedly different sets of standard deviation, although Fernández et al. [3] showed that a men: women ratio of 1:3 revealed gender differences. Third, we asked the participants to report only valence, arousal, and motivation as the indicators of emotional expressivity. However, this is inadequate. There are many other indicators such as facial expression, body language, wink reflect, and tone of voice. Combining all of this information rather than relying only on self-reporting might further elucidate the emotional expressivity. Finally, we measured only HR as the indicator of physiological responses. HR reflects only the activity of the sympathetic nervous system. Studies have shown differences between sympathetic and parasympathetic nervous system [29]. Future studies should adopt more indicators, such as galvanic skin responses and respiratory rate.

## Conclusions

The emotional responses elicited by emotional videos were inconsistent between emotional experience and emotional expressivity. Men had stronger emotional experiences, whereas women had stronger emotional expressivity. Gender differences in emotional experience and emotional expressivity depended on specific types of emotion, not only the valence.

## Supporting Information

S1 File All the data. (RAR)
